# Understanding sexual and reproductive health from the perspective of late adolescents in Northern Thailand: a phenomenological study

**DOI:** 10.1186/s12978-022-01528-1

**Published:** 2022-12-23

**Authors:** Panitsara Leekuan, Ros Kane, Panpimol Sukwong, Waratya Kulnitichai

**Affiliations:** 1 School of Nursing, University of Phayao, Phayao, Thailand; 2 School of Health and Social Care, University of Lincoln, Brayford Pool, Lincoln, LN6 7TS UK

## Abstract

**Background:**

Worldwide, Sexual Reproductive Health (SRH) issues comprise a third of health problems for women aged 15–44. SRH education equips people with knowledge of concepts around sexuality and reproduction, and the skills help to make informed decisions to prevent sexual and reproductive ill-health, including unplanned pregnancy and HIV/AIDS, and other sexually transmitted infections (STIs). The aim of this study was to explore the experiences of late adolescents relating to SRH, examining their attitudes toward sex and contraception, and to identify the gaps in knowledge pertiaing to decision-making around risk-taking behaviour.

**Methods:**

A qualitative phenomenological study was undertaken with 30 adolescents aged 18–19, purposively and snowball sampled from a university in Northern Thailand. Data collaction took place from July 2020 to January 2021. In-depth individual interviews were conducted until data saturation was reached. Data were recorded, transcribed, and analysed in ATLAS.ti version 9, using Modified Interpretative Phenomenological Analysis to identify pertinent themes.

**Results:**

Participants revealed five key experiences of SRH related to sex and contraception: *Keeping a secret*; *Seeking Freedom and Love*; *Having SRH education*; *Self-protection*; *Parental acceptance*. All findings reflected the value and impact of SRH on the experiences of late adolescents.

**Conclusions:**

This study provides detailed knowledge about adolescents’ perspectives of SRH and rights in terms of accessing sexual and reproductive health care and information as well as autonomy in sexual and reproductive decision-making. Gaining SRH education can assist decision-making concerning contraceptive methods for family planning and STI prevention. The study recommends that SRH and rights-based education should be designed responsively and appropriately for female and male adolescents, their families, and society. The content of SRH should be informed and advocated by healthcare providers, educators, policy makers, and systems to empower adolescents in order to achieve effective SRH education.

## Background

Adolescence is a period of transition from childhood to adulthood, in which individuals face profound physiological and psychological changes and challenges [1]. The period of adolescence can be frustrating; cognitive, physical and social development can be painful, traumatic, embarrassing, and unsatisfactory [2]. Although it can be a turbulent time, it is also a time of great potential, when young people develop more varied and complex relationships [3]. The major developmental changes that occur in adolescence have been well documented and have been grouped by a team from The US Office of Population Affairs and the Johns Hopkins University, into five overlapping and intersecting areas: physical (hormonal changes and development); cognitive (changes in the way the brain functions); emotional (how adolescents process emotions and stress); social (changes in familial, social, and romantic relationships) and morals and values (how adolescents regard their place in the world [4]. The onset of adolescent development brings new vulnerabilities, sometimes including human rights abuses, particularly in the areas of sexuality, marriage, and childbearing as many young people engage in new romantic and sexual relations and exposure to unplanned pregnancy and sexually transmitted infections [3]. Adolescents therefore face a range of health and social challenges as they mediate the biological and social transition into adulthood.

The experiences of late adolescents (aged 18–19 years) vary greatly by gender, race, ethnicity, socio-economic status, and other factors that shape how they respond to their physical and emotional development and assumption of the roles of adulthood. People commonly develop a deeper sense of identity during late adolescence, such as a personal sense of gender and sexuality, establishing values about sexual behaviour, and developing romantic relationships [5]. Embarking on sexually active relationships means the entrance to the world of ‘adulthood’ and potential parenthood. Lacking adequate knowledge and skills about developing safe sexual relationships can make adolescents vulnerable to a high risk of unintended pregnancy, unsafe abortion, and sexually transmitted infections (STIs), including HIV/AIDS [6].

Good sexual health not only includes the attainment of physical, emotional, mental, and social wellbeing about sexuality, but it also focuses on the absence of disease, dysfunction, or infirmity [7]. A positive and respectful approach to sexual relationships as well as the possibility of having pleasurable and safe sexual experiences, free of coercion, discrimination, and violence are key to sexual health and wellbeing [8]. Additionally, sexual rights which embrace certain human rights at the international and regional level, as well as national laws, must be respected, protected, and fulfilled, for all people, for the highest attainable standard of health [9, 10]. These are necessary conditions for attaining sexual health. Rights critical to the realisation of sexual health include the right to be free from discrimination; the right to privacy; the right not to be subjected to torture or ill-treatment; the right to determine the number and spacing of one’s children, and the right to be free from sexual violence [10, 11]. The World Health Organization (WHO) works to promote and protect everybody, including adolescents, to achieve their full potential for sex and reproductive health and wellbeing and to meet the needs of diverse populations, particularly the most vulnerable [12].

Sexual and reproductive health are closely linked [12]. Sexual health is complex and largely influenced by factors such as physical appearance, psychological factors, social factors, cultural norms and past experience [6, 7]. At the same time, the standard of health is crucial for the promotion of Sexual Reproductive Health and Rights (SRHR), including contraception, maternal and newborn health, and HIV/AIDS [13]. The WHO provides guidance and structure to sexual health programming and research, thereby supporting achievement of sexual and reproductive health targets [7]. In this regard, the International Conference on Population and Development, back in 1994, recognised  that reproductive health and rights, as well as women’s empowerment and gender equality, are cornerstones of population and development programmes and set out its own programme of action for adolescent sexual and reproductive health and rights (ASRHR) as well as service needs of adolescents. This heightened awareness that adolescent sexual health plays a vital part in reproductive health and well-being in later life [14]. The challenge remains in ensuring access to reproductive health care and education and addressing entrenched gender norms which continue to affect late adolescents as they manage the transition to adulthood [7, 12]. More importantly, accurate information can address gaps in knowledge, dispel misconceptions, build comprehensive understanding and foster empowering skills, positive attitudes and values, and healthy behaviours [6, 7, 12]. As comprehensive sex education finishes advancing adolescents’ knowledge, attitude and skills supportive of making informed sex choices and of building safe and respectful relationships, including awareness of cultural contexts, promoting mutually respectful attitudes between and among adolescents in connection with sex will form the foundation for the good health of populations as adolescents become adults, and for social and economic development more broadly [7, 12, 15].

Achieving good adolescent sexual and reproductive health (ASRH) in low and middle-income countries (LMICs) is a major public health challenge [6, 12]. WHO [6] reports that, worldwide, over 1 million adolescents contract a sexually transmitted infection (STI) every day. Globally, almost half of new HIV infections occur in people aged 15–24. STIs cause a huge health and economic burden, especially in LMICs where they account for 17 per cent of economic losses caused by ill-health. Adolescents are a high-risk group who face barriers to accessing accurate information about their health and rights and how to protect themselves from unwanted pregnancy and STIs [16]. Comprehensive sexuality education plays a crucial role in empowering young people to know and exercise their rights, including the right to delay marriage and the right to refuse unwanted sexual advances.

There are an estimated 580 million adolescent girls in the world today, of whom 88 per cent live in LMICs [9]. SRH continues to elude many, and many are denied the right to make safe and informed decisions that affect their health and wellbeing [17]. SRH is only one among the many dimensions of adolescent girls' health, notably including nutrition and mental health, and improvements in SRH depend on progress in other dimensions of health; at the same time, girls' SRH has huge implications both for their later health as well as the health of the next generation [17]. In light of the Unite Nation’s review of 20 years of implementation of the International Conference on Population and Development (ICPD) Programme of Action [18] and the global agenda for 2015 and beyond, it is important to review the situation of today’s adolescent girls and assess their need for sustained and expanded national and global attention and investment.

There are various public health concerns worldwide caused by high-risk behaviour among adolescents, such as early sexual initiation, multiple partners, and unprotected sexual intercourse. The United Nations Population Fund (UNFPA) reported a 25 per cent increase in global contraceptive prevalence worldwide [19]. As a result it is internationally recognised that the adolescent birth rate has decreased steeply and the maternal mortality ratio has declined. However, improvements have been slow and have varied by country, and an estimated 100 million women worldwide are still not using safe and effective contraceptive methods to prevent unwanted and unintended pregnancy and a new global target (SDG 3.1) on reducing maternal death has not been met [12]. One of five critical targets to help countries in reducing preventable maternal deaths is for 65 per cent of women to be able to make informed and empowered decisions regarding sexual relations, contraception use, and their reproductive health. Therefore, urgent action is needed to improve the health and survival of women and infants [12].

Thailand, as an upper-middle-income economy, is faced with a growing number of adolescent pregnancies and an increasing rate of STIs, including HIV/AIDS [20]. These statistics have been attributed to the consistently falling prevalence of contraceptive use [20]. The national survey on the prevalence of consistent condom use at last sex among young people and adults aged 15–24 years found that only 60 per cent had consistently used condoms [21]. A study [22] in Thailand revealed that 75.8 per cent of secondary school adolescents used contraception during sexual intercourse. Of these, 84 per cent consistently used condoms. Contraceptive use among unmarried school-going adolescents is crucial in maintaining a stable balance between their sexual and reproductive health. Therefore, adolescents could be encouraged and supported to use contraception and prepared emotionally to practice safe sex. Typically, young people are fully developed in their sexual identity by this stage [23]. They may also live independently from their families and may take on adult responsibilities and roles [24]. Late adolescents are able to understand the consequences of current actions and are often concerned about their future, and career goals, and may be considering desirable potential spouses or life-partners related to transition to the adult role [23, 25].

Adolescents are a heterogeneous group with different and evolving needs and  highly susceptible to social pressure depending on their development stage and life circumstances. As they  move from adolescence into adulthood, they must be prepared to face the challenges encountered in the adult world.

Given the many physical, sexual, cognitive, social, and emotional changes that happen during adolescence, it has been acknowledged that understanding what to expect at different stages can promote healthy development into early adulthood and the following different stages of adolescence have been suggested: early adolescence (ages 10–13), middle adolescence (ages 14–17) and late adolescence (ages 18 +), [26] the latter group being the focus of this current study. We have focused on late adolescents because of the likelihood that they have increased cognitive ability and psychological developments that allow clear decision-making regarding participation and the discussion of sensitive topics [27]. They usually have more impulse control by this point and may be better able to gauge risks and rewards accurately They also face novel challenges, including engagement in romantic and sexual relationships [28]. It is argued that late adolescents develop their own individuality further than younger adolescents and this can boost self-esteem and confidence. They may become more concerned about the future and thought about their roles in life. However, many reconcile a particularly strong relationship with their families to seek advice and support [26].

Globally, there has been an increasing number of sexually active adolescents [6]. Initiation into sexual activity is a part of normal behaviour and development but may be associated with negative outcomes if sexual behaviour involves engagement in sexual activity at a very early age or without attention to risk behaviour. Adolescents may face many sexual and reproductive health risks stemming from early, unprotected, or unwanted sexual activity. Adolescents therefore need protection from harm on the one hand, and support to make independent decisions and act on them on the other.

The contraceptive needs of adolescents are varied and changing. When adolescents are able to obtain and use contraceptives, they face barriers that prevent their use, or consistent and correct use, including pressure to have children, stigmas surrounding non-marital sexual activity or contraceptive use, fear of side-effects, lack of knowledge on their correct use, and factors contributing to cessation. Indeed, the unmet need for contraception is higher among adolescents than in any other age group.

Previous studies have explored the experiences of pregnant adolescents and revealed that contraceptive decision-making is influenced by the quality and type of sexual health education, especially knowledge about contraception and adolescents’ perceptions regarding hormonal contraception as well as gender-based power imbalances in intimate relationships [29–31]. Gender roles and imbalances of power can influence the outcomes of adolescent sexual activity including pregnancy when girls lack the negotiation power to insist on contraception use [1, 19, 30, 32]. The influence of gender roles on sexual and reproductive health may be presented in terms of existing gender power imbalance and the assumption that males have higher social status than females [30, 33]. Congruent with previous studies, female adolescents have been pressured by their partners into obeying their partners’contraceptive preferences and to have sexual intercourse without the use of contraception [33, 34]. Gendered imbalances of power have been shown to result in unmet contraceptive through the influence of differential gender roles in determining the lack of contraceptive use [30, 33].

One view is that young women are inclined to engage in sexual activity to show love or establish long-term relationships, but young men typically engaged in sexual relationships for curiosity or the need to satisfy their sexual drive [35]. Moreover, the adverse consequences of sexual activity for young women include unwanted pregnancy, sexual violence, or partner abandonment. Young women can also experience unequal gender relations which can negatively influence their sexual health [1, 30, 34].

To be effective, adolescent men should be central to interventions and educational programmes about SRE including those which address men’s behaviours in their various roles as well as their reproductive health and rights as human beings [12, 36]. Young men do not always receive comprehensive sexual health education including prevention messages and information about how to access services [6]. In order to prevent pregnancy and reduce the risks of STIs and HIV/AIDS, accessing and using contraception enables adolescents to exercise their rights to decide freely and responsibly the number and spacing their children, and to have the information, education and means to do so [6, 12]. Enhancing sexual reproductive health education is therefore vital among late adolescents, as this can assist in preventing adverse outcomes of sexual relationships [6]. Understanding sexual reproductive health experience and its impacts on practices among late adolescents is crucial for promoting sexual reproductive health and rights in the transition to adulthood as well as opportunities for improving sexual and reproductive health care services and interventions [12].

Due to the private and sensitive nature of the subject, relatively little is known about SRH among late adolescents, particularly in relation to their knowledge, attitudes, and practices, and this is also the case in Northern Thailand. To address this gap and to provide data for improving the understanding of SRH experience and also to inform prevention messages and services delivered to female and male university students, we conducted an interpretive phenomenological study to better understand and describe late adolescents’ experience of SRH. The study was also undertaken to generate evidence relevant to a wide range of SRH programmes for adolescents and practitioners.

## Methods

Reporting adheres to the Consolidated Criteria for Reporting Qualitative Research (COREQ).

### The aim

The aim of this study was to explore the experiences of late adolescents relating to sexual reproductive health, examining their attitudes toward sex and contraception, and to identify factors influencing decision-making to help to prevent future risk-taking behaviour.

### Study design

Interpretative phenomenology, as developed by Heidegger [37, 38], was selected as the research methodology in order to answer research question: “*How do late adolescents understand Sexual and Reproductive Health*?”. This approach is effective in bringing out what is usually hidden human experience. In interpretative phenomenology, the expert knowledge on the part of the researcher is valuable in the interpretation of the narratives provided by the participants. This method goes beyond the descriptive in looking for the meaning hidden in the narratives and assists in the understanding of how individuals make sense of their SRH experience.

### Study setting

The study was conducted in a university in a province in Northern Thailand. In line with phenomenological study, the sampling was designed purposively to capture the experiences of adolescents who met specific inclusion criteria, including those who had had experience of sexual intercourse. The study included adolescents aged 18–19 years (referred as late adolescents), who, in comparison with early and middle adolescents, tend to be more composed and mature, having generally already acquired major physical changes and cognitive maturity. Adolescent features such as risk-taking, curiosity and anxiety are less prevalent among late adolescents [1, 39].

University students are currently pursuing higher education and have been through late adolescent development into early adulthood or middle adulthood [40, 41]. University students also tend to have emotional stability and more critical thinking than their younger counterparts. In Arnett’s emerging adulthood theory [42], university students with an age range of 18–19 years are generally still in the process of finding their identity as evidenced by being still in the study period, still looking for a permanent job, and not fully independent from their parents. In this context, university students have higher demands and increasingly difficult assignments as well as stress triggers.

To recruit participants, this study employed two different kinds of sampling: purposive and snowball. For the phenomenological approach, purposive sampling was suitable because the goal of this study was to examine the experience of adolescents who were sexually active. Snowball sampling involves collecting data from a few participants from the target population who can be reached initially, then asking those participants to help recruit others from their social networks. This is appropriate when the target population are hard to reach or difficult to locate, as these participants had proven to be. Sampling was terminated at the point of redundancy when no new information was forthcoming from participants.

### Data collection

Two volunteers who worked in the university student services department assisted in recruitment by contacting eligible participants and providing the relevant research documentation/information via email and then referred potential participants to the principal researcher (LP). Initially 5 individuals expressed an interest in taking part and the principal researcher (LP), in line with the purposive sampling strategy, checked their compatibility with the inclusion criteria, ascertaining that they: (a) were 18–19 years of age, (b) had had heterosexual intercourse, (c) were able to communicate effectively in the Thai language (d) were willing to be interviewed and audio- recorded, and (e) were able and willing to provide informed consent. The only exclusion criterion was: (a) they had left the university during the study. The principal researcher (LP) made an appointment with each participant prior to the interview.

Data were collected between July 2020 and January 2021. An unstructured individual in-depth interview with each participant (lasting 60–90 minutes), was conducted by the principal researcher (LP). Due to the COVID-19 pandemic, interviews were held remotely, via Zoom Cloud Meetings which allowed visual interaction and personal communication. In line with phenomenological study, both open-ended and flexible questions were included to facilitate participants’ description of their everyday lived experience. Prompts such as “*Could you please tell me more about…?*” Or “*Could you please give an example of….?*” were used to encourage the adolescents to elaborate. During the interview, behavioral and non-verbal cues were recorded in the principal researcher’s field notes and where appropriate, probed for further clarity from the participants about how they were feeling. Field notes were written up following the interviews and were structured into four parts: observational notes (what happened), theoretical notes (deriving meaning as the researcher reflected), methodological notes (critiques, instructions, or remainders to oneself about the research process), and personal notes (summary analytical memos) in order to facilitate reflection on the interviews.

The principal researcher (LP) paid attention to “bracketing” her ideas and assumptions. In order to bracket the researcher’s preconceptions, and not influence the study findings, the principal researcher (LP) wrote down her own understanding of sexual reproductive health and related experiences.

The participants were interviewed at their preferred time to maximise privacy and confidentiality. The principal researcher (LP) initiated a warm-up discussion to help the participants to relax and reduce any tension between the participants and the researcher. Interviews were conducted in the Thai language and all were audio-recorded.

At the end of each interview, the principal researcher (LP) thanked the participant and asked her/ him to help recruit further participants (whom they felt might meet the inclusion criteria) from their social network. Thus, participants continued to be recruited through snowball sampling. Data were collected until analytical saturation was reached and new insights stopped emerging, after 30 participants had been interviewed.

### Data analysis

Initial data analysis took place alongside data collection. The principal researcher (LP) interpreted individual experiences from what the participants had said about their experiences, and what appeared to be important to them. She was actively interpreting the responses from the participants, whilst maintaining reflexive diary notes during the interview, while also being keen to maintain rapport and engagement with interviewees. Upon completion of the interview, the principal researcher (LP) recorded her overall understanding of each interview in order to preserve the immediate sense of the experience of the participants.

Next, in order to immerse herself deeply in the interviews and thought process, the principal researcher (LP) transcribed the interview data in Thai and anonymised the transcript (in terms of the names and other identifying information pertaining to participants and their associates). The principal researcher (LP) listened to the audio recordings of each interview three times each, with the first time being immediately after the interview in order to achieve familiarisation used the reflexive diary to improve recall and reduce recollection inaccuracies. These practices greatly contributed to data immersion and enabled the subsequent identification of emergent themes with a comprehensive sense of the whole: The principal researcher (LP)’s interpretation and understanding of late adolescents’ experiences was derived from the process of constructing the transcripts by listening and re-listening to the interviews.

The analysis continued via a process of discussions between the interpretive team (SP, KW) in order to follow the Heideggerian principle. To describe the experiences of SRH among late adolescents, the principal researcher (LP) analysed the data following Modified Interpretative Phenomenological Analysis outlined by Heidegger [37], Packer [43, 44], van Manen [45] and Smith et al. [46]. The analysis was done via the following steps: (a) initial read and re-read of each interview transcript and listening to the audio- recordings in order to achieve data immersion and identify meaning units [45]; (b) highlighting the interpretive processes and assigning codes or phases; (C) the coding meaning units were merged and developed into sub-themes concerning the same concepts relating to particular phenomena: (d) similar sub-themes were identified links and developed as well as refined to themes in order to interpret participants’ experiences; (e) member check to clarified disagreements in interpretation; (f) eliciting responses and suggestions on a final draft from interpret team. While performing each of the steps carefully, moving between all steps was done iteratively, from the overall impression to particular parts of the transcript, identifying themes and subthemes of late adolescents’ perspectives. The analysed data were translated to English at the point of publication.

### Rigour of the study

The research team was mindful of the concepts of credibility, dependability, confirmability and transferability [47], and their importance for increasing the trustworthiness of this study.

*Credibility* was guaranteed by using purposive and snowball sampling to select participants who had direct experiences of the phenomena under study and were willing to share their stories. The development of the relationship between the researcher and the participant throughout the duration of the interview also helped to build trust. Member checks were undertaken, this involved sending each transcript back to the participant and inviting them to correct any errors or misinterpretation.

*Dependability* involved having a researcher outside of the data collection and data analysis evaluate the analysed data to ensure that nothing was missing. Data from several data collection processes such as interviews, observations and written field notes were used and all analytical data were linked to aid understanding of the phenomena of SRH in late adolescence.

*Confirmability*, a reflexive journal for the researcher self-awareness was written before and immediately after the interviews and notes were taken during the interviews, to reduce bias. A reflexive diary captured the principal researcher’s (LP) thoughts, feelings and ideas during the study process, and was used to inform the development of the research process and allow the reader to critically appraise the quality of the study. When reading this manuscript, this transparency will allow the readers to decide upon their own interpretation of what the researchers have revealed, and then apply a fore-structure of understanding of this study. Two specialists in qualitative research also audited all research processes and document as well as the tentative findings.

*Transferability* the reader should be able to transfer the information gleaned from the study and find it meaningful and applicable to their own experience. Therefore, this is intrinsically not transferable. While conducting this study the principal researcher (LP) found that some experiences shared by participants were similar to those she had encountered from other late adolescents before beginning the research. This similarity was justified. The experiences shared by participants with the principal researcher (LP) may apply to other late adolescents (Table 1).

**Table 1:** Coding process –meaning units, sub-themes and main themes

Meaning units	Sub-themes	Themes
I remembered that condoms could prevent sexual diseases. I disliked taking pills and feared implants, and contraceptive injections. However, if I didn’t use any contraceptive methods, I would face unplanned pregnancy and be out of schooling. I don’t want to stop schooling due to pregnancy. We might face with some problems, such as raising children and finance.* [RH 18]*	*Focusing on the risk of diseases and consequences*	Having SRH education
When I had sexual intercourse and used condoms, there was an accident of a broken condom. I felt fear and stress. I did not know how I could solve this problem. I then took emergency pills at first. I also waited for my period. After that, it came. I therefore felt relieved. [RH *25]*	*Using contraceptive methods*	Self-protection
*My partner asked for having sexual intercourse with me. I then asked him about condoms. He drank alcohol and did not prepare a condom. I said “no”. He was angry. I reasserted my choice and tried to explain more about the negatives of non-use of any contraceptive methods. If we made the mistake, we might have some problems. [RH 26]*	*Negotiation*	

## Results

The participants in this study comprised 30 adolescents (3 men and 27 women). 17 were aged 18 years and 13 were aged 19 years. In terms of marital status, 14 participants were co-habiting with their partners and 16 were single.

The five identified themes reflected the phenomenological interpretation of late adolescents’ experiences of SRH: *Keeping a secret*, *Seeking freedom and love*, *Having SRH education*, *Self-protection*, and *Parental acceptance.*

### Keeping a secret

Adolescent engagement in a sexual relationship with someone of the opposite sex caused disapproval from some parents and families. Many participants contrived not to tell their parents or families of their relationships with their partners. A simple reason for secrecy was the feeling of unease in exposing their relationships for fear of this resulting in parental disappointment.

*My dad didn’t know about my relationship with my partner. This was because he didn’t want me to have a boyfriend. He also wanted me to graduate studying before having a boyfriend because he worried about a social stigma. My dad had more authority to decide everything in my family. I feared him when I had to ask him for permission. I was scared of him the most. I kept my relationship as a secret. He also asked me “What would you select between studying or having a boyfriend” I answered that I chose studying.* [RH 21]

*My mom told me that I should finish my studying rather than have a boyfriend. She worried about my studying. My parents didn’t permit me to have a boyfriend. My dad is very strict. I had a boyfriend but he did not know about this. I then kept it as a secret until I graduated.* [RH 20]

### Seeking freedom and love

Most participants in this study searched for freedom from parental strict rules and regulations. They felt excited when they left their houses for their educational setting. This seemed to offer a new world for them to seek new friends and a new environment around them. Some participants described their personal encounters in seeking love and an act of love on their part. When they felt close to partners, participants reported feeling happier and even healthier. These participants felt loved, understood, and safe in their new relationship:

*Our first relationship took place when he bought some food and brought it to me. He also took care of me as well. Nowadays, we live in a flat outside the university, but we are living separately. Our flat are opposite. However, he often comes to see me and pick me up for studying. This makes me feel that I receive the love from him all time that I try to find it. I think that it is giving love together. I also appreciate with our relationship and his activities.* [RH 03]

*We confide to each other. When I cannot decision by myself, I often openly talk with him and ask him for suggestions. He always gives me about benefit ideas. I think I am so lucky to have him as my boyfriend and I got love from him that I seek.* [RH 16]

#### Intimate relationships

An intimate relationship, including physical, emotional, and sexual intimacy, can lead to having full sexual intercourse. Among the reasons for having a sexually active relationship, adolescents sought love, acceptance, and recognition. The majority of female participants expressed a desire to be worthy of recognition and love, and they believed that living together and having a sexual relationship could maintain their union with their partners.

*Our relationship seems like a close friend relationship. He is a friend who can discuss every story with me. He also took care of me and his concern has always been about my activities. This made me feel that there was someone to care for me and love, apart from my parents. I felt better with my life. I also did not worry about his behaviour. I was then happy and studied efficiently.* [RH 01]

Most participants reported that intimacy promoted feelings of endearment when their partners took care of them. This also reflected feelings of love and trust, honesty, and the comfort that came with having intimacy.

*I felt that he loved me when he took care of me and sent me to study every morning. We love together. I think that is giving love to each other. We had only time to live together, chat and confide in someone. This was my happiness.* [RH 03]

*In my opinion, living together and having sexual relationships are normal. Nowadays, these behaviours are accepted. Expressing love through intimate sexual relationship increases emotional attachment.* [RH 20]

#### Openly sharing feelings

Building and maintaining intimacy in a relationship depends on open communication about feelings and desires. This also includes sharing thoughts and feelings openly to raise the level of emotional intimacy. Participants exposed how they felt during cohabitation and having sexual relationships.

*I am an extrovert. It was not wrong if we could talk openly together. Finally, we accepted each other. In the initial time, we did not dare to talk face-to-face. I sometimes felt pain when we had sexual activity. I therefore told him. I think that non-verbal communication is not good, because others do not know how you think and what you want. We should learn about each other.* [RH 03]

*My boyfriend was willing to talk about difficult issues and to find a solution together. I trust him because I can tell him everything in my life. He also took care of me. We respected each other. This made our relationship strong. I always feel better when we can think things through together* [RH 28].

### Having SRH education

The majority of participants perceived that SRH education was important for learning and good health, as well as for decision-making to use appropriate contraceptive methods. SRH education also assists in controlling behaviours and reiterates the deficiency of correct and easily accessible SRH information sources. Participants indicated that they learned about contraception from school, the internet, friends, family, and health providers, but schools, universities, and health providers were the most frequently cited.

*During studying in secondary school and high school, I attended a training programme specially designed for adolescents about SRH education. It plays a crucial role for youth and is compulsory in the schools. I also learned about the risk of sexual transmitted diseases and HIV/AIDS and contraception in General Education in the University. Additionally, I saw it in the movies and on YouTube and in any video clip about the risk of sexual transmitted diseases and HIV/AIDS, and prevention from unintended pregnancy. As a result, this can increase my knowledge about the results of sexual relationship problems and brush up, as well as repeat warnings about preventing these diseases. I believe that studying provides new knowledge, even though it is repeated again.* [RH 06]

*I heard about contraception methods since I studied in the secondary school until in the University. All information involved in the risk of sexual transmitted diseases and HIV/AIDS and using contraception.* [RH 09]

SRH was mainly discussed in terms of the risk of diseases and consequences of not using contraceptive methods, particularly STIs. SRH education influenced contraceptive decisions, due to the desire to prevent STIs and consequences such as dropping out of schooling and financial difficulties due to unplanned pregnancy. These participants stated that they obtained SRH education related to the risk and the consequences of unprotected sexual intercourse from many sources.

*In secondary school, some health providers from a hospital provided me with education about contraceptive methods, such as the contraceptive implant, condoms, and oral pills, as well as protection from the risk of sexual relationships. I also studied in General Education about the risk of sexual transmitted diseases and HIV/AIDS and contraceptive methods, as well as the risk of having sexual relationships on special occasions such as Valentine’s Day or Loy Kra Thong’s Day.* [RH 01]

*I got free condoms from health providers from a hospital. They provided knowledge of contraception and protection from the risks of sexual transmitted diseases. I was shame at the first. But the next time, I felt better after I knew that there were many methods to protect from unplanned adolescent pregnancy and diseases. Condom was a method to prevent from the risks of transmitted diseases.* [RH 08]

### Self-protection

Having SRH education in adolescence can increase awareness about prevention from the risk of STIs by using contraceptive methods. The awareness about different contraceptive methods, including condoms, hormonal methods (i.e., oral pills, implants, and contraceptive injections), can assist adolescents to suitably utilise choices for them and their partners.

#### Using contraceptive methods

Both male and female participants revealed that the most commonly used contraceptive methods were male condoms and oral contraceptive pills. Some of them had experienced accidents (e.g., a broken male condom), as a result of which they used combined approaches (i.e., emergency oral pills).

*I asked my partner to use a condom when we had sexual relationship. I sometimes bought oral pills if he did not prepare male condoms. I think that protection from the risk of sexual transmitted diseases and HIV/AIDS is a crucial issue for us, me and my partner. We have love and must protect ourselves* [RH 02]

*I lived together with my boyfriend. I took oral pills and used condoms. I bought male condoms for my boyfriend. One night, we did not use any contraceptive methods. We were frightened and worried about the result of  our ignorance. I then decided to toke emergency pills. After that, I never forget to protect myself during sexual relationships.* [RH 14]

#### Negotiation

Female participants disclosed that they negotiated condom use prior to having sexual activity, in order to achieve safe sex. Their priority was preventing the risk of STIs and unintended pregnancy.

*I always ask him before having sex about condom. When he didn’t have it, I said “No”. Although he tried to persuade me, I confirmed the same word. He then felt frustrated and went back to his house. I refused him when he requested to have sexual relations without using a condom. He accepted my decision.* [RH 03]

*When my boyfriend did not use a condom, I was not okay. He drank and felt angry. I confirmed that I was frightened and concerns about unintended pregnancy. Later, we talked together and I gave the reasons to him why I refused him. He listened to me and promised that he would use a condom.* [RH 25]

#### Concern for the family

To address concern for their families, participants attempted to avoid unintended pregnancy and the risk of sexual transmitted infections. The vast majority of participants attended sexuality education and so they were keenly aware of the potential lifelong consequences of unprotected sexual activity.

*It is not only that I love him, but also there are many people in our families who love us. If I was ill or pregnant due to having sex without protection, they would regret it. I am also a university student; therefore, I am not ready to be a housewife or a mother. I think of my parents. They raised two daughters, and they might not want to have a grandchild at this time. We might have financial problems.* [RH 02]

*Young people have to take responsibility. For me, I think that it is very important. If I cannot take responsibility for safe sex and become a pregnant woman, this will become parents’ duty to raise a child in the future. This can make my parents disappointed in me. So, I am aware of the ignorance of not using control birth methods*. [RH 23]

#### Parental acceptance

Premarital sex is common practice among young people in many countries, including Thailand. This pattern varies substantially across generations. Rapid change in Thai society and culture is reflected in changing sexual values and mores. The family, school, and society view unplanned pregnancy, abortion, and sexualised media with disapprobation. The family is a significant factor in the development of sexual behaviour among adolescents. Participants described their own parents and partners' parental approaches to their relationships. The participants described how that both families had opportunities to get to know each other, as a result of which they now trust their children.

*My parents had expectations about my future because I was the oldest daughter. My parents therefore wanted me to grow up as a good adult in society and to prevent unplanned pregnancy. Initially, my mother did not approve about our relationship. We met together via Facebook around a year before becoming lovers. After that, he and his parents came to see my family. Both families accepted our relationship.* [RH 01]

*I decided to expose my relationship with my boyfriend to my dad. He said that I was okay and wanted to meet him. He did not forbid me to have a boyfriend. He trusts me because I have never been able to disappoint him.* [RH 29]

#### Openness

Adolescents had perceptions of the openness of family communication and the significance of subjective meaning in communication and relationships. Some adolescents who perceived their communications as open and problem-free with their mothers considered that this influenced their decision-making about taking care for themselves and preventing sexual health problems.

*I felt better when I told my parents everything openly, and they listened to my voice, such as private stories or studying. This has taken place from the past until now. Therefore, when we faced some problems, our parents gave some suggestions. My parents were initially concerned about my relationships with my partner. My mother told me that you should rethink about the relationship, but she did not blame me. She always tells me about caring for myself and protecting from the risks of sexual activities, such as unintended pregnancy and diseases.* [RH 17]

*My mom is a kind person. She understands about adolescent life. She never forbids me to have a boyfriend but told me that you should take care of myself. I then told the truth to her about our relationship.* [RH 20]

#### Trustfulness

Trustfulness refers to beliefs about  the honesty and reliability of others, including their decisions and actions. Trustfulness also provides a positive basis for relationships. The participants described their mothers who believed in their behaviours and decisions about their relationship with their partners.

*I and my mother always talk about every story together. I told her that I had a boyfriend. She gave me many suggestions. When we have some problems, she then tried to come down. She knew about our relationship and us having a sexual relationship. However, she did not blame me, but she suggested me to use contraception. She also motivated me to look ahead.* [RH 20]

*My mom didn’t want me to become a pregnant woman before I graduated. She knew about my relationship with a boyfriend. She also told me to protect myself from pregnancy. I did follow my mom’s suggestions. She then trusted me because I was aware of the consequences of the absence of using contraception.* [RH 23]

## Discussion

### Adolescent love and romantic relationships

Making sense of their experiences, the participants in this study revealed how they sought love. They had also looked elsewhere for satisfying relationships and thinking that they had found love, their behaviours with their partners involved sexual intimacy. The experiences of seeking love and romantic relationships identified in the current study were broadly consistent with developmental theories [48, 49]. Previous studies on adolescent love and romantic relationships have found that those in middle to late adolescence worry about maintaining and sustaining relationships and intimacy levels, and emotional investment, skills and commitment in romantic experiences increase across all developmental stages of adolescence [49].

Adolescent romantic relationships can not only improve growth, resilience, and happiness, but can also constitute sexual development which is an important part of growing to adulthood [25]. Most participants believed that having boyfriend or girlfriend can enhance one’s confidence. They were also happier in themselves with the support, trust, and closeness found in their romantic relationship. Similarly, qualities of adolescent romantic relationships can influence positive mental health development into adulthood [50]. This mirrors findings of the link between supportive partners and increased mental health development.

Romantic relationships are an important aspect of individual development and socialisation, especially in adolescence [25]. Through romantic relationships, individuals learn intimacy and crucial interpersonal skills. Romantic relationships can play a positive role in adolescent development [51], including  psychological development [52, 53].

Adolescents commonly experience their first romantic relationships and may experience overpowering emotions associated with falling in love, which can lead to having sexual relationships despite rational objections to this. Relationships in which sexual activity is the primary aspect can also arise, such as transactional sexual encounters, which have can have serious socio-economic, sexual, and psychological impacts. This study illustrated the feelings of romantic love associated with enhancing happiness and life satisfaction in adolescent relationships with increasing maturity. Wheeler et al. [54] reinforced that the nature of romantic relationships during late adolescence and young adulthood can shed light on many aspects of adolescent development, including the distinctive developmental contexts of intimate relationships. Similarly, Kansky and Allen [50] revealed a link between romantic relationships with supportive partners and future intimate relationships and wellbeing. Adolescent romantic relationships have the potential to affect psychological functioning well into adulthood [50]. Connolly and McIsaac [25] verified that adolescent romantic encounters are initial steps on an experience toward a mature relationship to adulthood. As a result of these effects, romantic relationships can cause adolescents to feel or to be perceived as more mature, with enhanced responsibilities and self-image, and future expectations, including with regard to long-term relationships [55, 56]. However, earlier studies found that adolescent intimate relationships with partners come with other risks, such as increased risk of STIs and unplanned pregnancy [30, 57].

Adolescents’ romantic relationships provide an opportunity of establishing and maintaining intimate relationships and developing positive effect on growth individual development [58]. They can also assist adolescents in developing their self-esteem and improving self-worth which grows through relationships with others [58, 59]. Having romantic feelings and engaging in romantic relationships are significant indicators of adolescent development [58]. However, adolescent romance may bring benefits to their growth but also lead to early sexual behaviour and serious SRH problems if adolescents are unprotected.

Romantic relationships which are characterised by intimacy and good communication can contribute to healthy adolescent growth, resilience, and happiness, and often provide a valuable foundation for long-term relationships into adulthood. However, while they are associated with healthy, normative development in most adolescents, they can be symptomatic of pathology in many others. Adolescent romantic relationships play an important role for the development of adolescence and can influence their future  wellbeing [25, 55].

### The power of SRH education

Participants in our study mainly learned about issues related to SRH such as puberty, contraception, sexual intercourse, becoming pregnant, and abstinence, from school, healthcare professionals, or the internet. A study of adolescent students by Deshmukh and Chaniana [61] also showed  the most common sources of information about SRH to be  teachers, mass media, and friends, while parents and siblings had the least important role. The role of school as a source of learning about SRH mainly relates to sex education/ health classes, which vary according to the age of the learners, cultural expectations, and national policies. Various forms of sex education are commonly offered from the early years of elementary school in Western countries, while in most Asian countries it is provided at the high school level. Among university students, participants reported that they received reiterations of SRH education with which they were familiar from their high schools.

SRH education axiomatically plays an important role in sexuality education programmes; in particular, it renders helpful knowledge and shapes attitudes towards human development, sexuality, relationships, gender roles, and decision-making about sexuality [57]. It also breaks down barriers in the prevention of adolescent pregnancy [62]. The integration of sexuality education in the curriculum provides a broad and rich channel of information about a variety of sexuality-related issues, from the growth and development of the human body and reproductive physiology to the development of healthy sexual attitudes and values.

Deshmukh and Chaniana [61] reported that very few parents were prepared to discuss reproductive health-related matters with their children at home, commonly due to a lack of awareness of reproductive health (60.75%), and fear about encouraging premarital sex (51.40%), aside from incidental shyness and stigma associated with sociocultural norms about discussing sexual issues with their own children. Notwithstanding, the current study found that parents openly communicated with their children concerning safe sex which influenced decision-making in contraception use. Indeed, parental communication is a crucial and effective tool for promoting healthy and safe sexual practices [60, 61].

This study also showed that most participants obtained SRH education in high school and university, which they considered important for their sexual health and decision-making concerning contraceptive methods. Over the course of many years of gathering knowledge of SRH, they perceived benefits of SRH education to include better and more accurate information and a better understanding of body changes, alongside empowerment with skills such as negotiation (particularly for females), decision making, and communication, in order to protect themselves from unintended pregnancy and STIs. This is in line with a study of Vongxay, Albers [63], which concluded that comprehensive sexual education and enabling information, as well as service access for adolescents, is essential to ensure that adolescents can access, understand, appraise, and apply good SRH knowledge in decision-making for optimum personal health. Oonyu [64] supported this and determined that SRH education was necessary for female university undergraduate students in Uganda.

The accuracy of SRH education was reported to be particularly important among participants in shaping their sexual relationship choices and consistency of contraception use, consistent with previous studies which revealed that adolescent attitudes toward contraception and the accuracy of condom and reproductive knowledge directly influence contraceptive use throughout adulthood [65, 66]. Conversely, Yared, Sahile [67] found that over half of university students in central Ethiopia knew about STIs but neglected to apply their knowledge to themselves and their sexual health. The majority of reproductive health problems in their study related to unwanted pregnancy and abortion caused by not using contraception. They thus suggested that SRH education should begin from high school, due to the fact that the majority of students in high school started sexual experience at an early age. Similarly, a study of Oonyu [64] in Uganda revealed that the majority of university students (66.3%) requested SRH education to assist them to overcome barriers, such as an inability to get reliable and accurate information to empower them in decision making, and to overcome inadequate education from parents and the existing university provision.

The lack of accurate information coupled with low access to contraceptives may increase the risk of STIs, unintended pregnancy, and other health consequences [66, 68]. Comprehensive SRH education and counselling could improve effective contraceptive behaviour throughout the lifecourse [65]. Therefore, SRH education is necessary for university students to resolve decisions about relationships, sexuality, and sexual behaviours [64]. However, the information transmitted about STIs and SRH via the media is commonly found to be boring by adolescents [67]. It is suggested that such programmes should be youth-friendly, with separate packages targeted to STIs and SRH.

Previous research indicates that perceived risks of pregnancy and STIs and motivation to avoid these outcomes are linked with adolescents’ contraceptive method use [64, 65]. In this study, late adolescents perceived that the risks of pregnancy and STIs were associated with contraceptive use. This reflects cognitive development, whereby late adolescents link current behaviours with potential future consequences more strongly than younger adolescents and children. Morales, Vallejo-Medina [69] reinforced that adolescents more knowledgeable and displayed more favourable attitudes towards different aspects of HIV/AIDS. Adolescents who perceived greater risk and negative expectations about risky sex outcomes related to pregnancy and STI were more strongly influenced in their final decision to use dual contraceptive methods [65, 66]. Adolescents’ attitudes about practical, social, and moral implications of using birth control are also associated with their final protective decisions [69].

Adolescents need and have a right to SRH information and services [70]. Providing SRH education in school and University is a cost-effective way of reaching adolescents [71]. Having SRH education and enabling information for adolescents are essential to help adolescents adopt safe and responsible practices in decision-making to benefit their own health [72]. The capacity of adolescents to make informed decisions based on correct knowledge on SRH is one of factors that contribute to the prevention of SRH problems, especially adolescent pregnancy, the risks of STIs and negative social consequences [63]. Therefore, accessing comprehensive sexual education should be provided for every stage of adolescence in order to inform decision-making for right way to contribute to SRH [63]. This is also the process of individual adolescents’ thought on an SRH problem before taking an action.

SRH covers many issues related to the reproductive system and its function. Sexual health indicates the ability to have a safe and satisfying sex life [13] and is also a human right and an achievement of the previous UN Millennium Development Goals. SRH education is an influencing factor for adolescent pregnancy, child marriage, and other adverse SRH outcomes and negative social consequences, which occur more often in poor, less-educated, and rural communities such as in Northern Thailand [73]. This study exposed the connection of SRH education and making decisions about having sex safely. Therefore, SRH education should occur throughout the school journey, with information appropriate to students’ age, religion, and cultural background. It should go beyond the current focus on biological aspects of sex and reproduction, and incorporate communities’ attitudes, values, and skills.

Aside from schools and universities, SRH knowledge from parents was crucial in sexual education. Parents are the largest influence on their adolescents’ decisions about sex, and they have a profound impact on those decisions. Participants in this study stated their parents told them to take care of themselves and prevent themselves from the risks of having sexual relationships. The prospect of talking about topics related to sexuality creates anxiety and apprehension, and this may lead to avoidance of such discussions [74]. This underscores the critical role of educational access for the realisation of SRH and rights for young people, especially girls [75].

Every adolescent has life-changing decisions to make about their SRH. The study illustrated critically important for providing sexual education to adolescents in order to improve adolescents’ SRH. As adolescents are at risk of adolescent pregnancy and their elevated risk of STIs, including HIV/AIDS, universal accessibility of information on SRHR plays a crucial role in the quality of life for everyone [2, 76]. Globally, as adolescents face with the double injustice of having limited access to SRHR information and services, several organisations working on adolescent and young people, have formulated guidelines to assist in creating curricula and examining previous curricula to expand knowledge for youth [12]. Thus, improving SRH and rights is an important for adolescents in the next generation. Adolescents who have access to SRH education are better supported on their way to a healthier future and can benefit from having corrected knowledge resulted in decision- making to use contraceptive methods for protecting themselves as well as having positive impact on the family and the community at large [12, 76].

However, the lack of sexuality education to empower responsible decision-making can predispose adolescents to unintended adolescent pregnancy and STIs and leave females vulnerable to coercion [19]. Lack of awareness of contraceptive use is also strongly associated with increased risk of early pregnancy. Disadvantaged adolescents are more inconsistent in their use of contraception, even when they do not want a pregnancy, and they tend to have more unintended pregnancies. A study in Thailand by Chirawatkul, Rungreangkulkij [77] found that adolescent women who achieved well in school and did not exhibit risky behaviours were likely to become pregnant when they do not have sufficient knowledge and skills to protect themselves from such behaviours, and that adolescent men had low understanding of how contraceptive methods work. Adolescents’ decisions and behaviours can have long-lasting implications, and being ill-informed can have lifelong adverse consequences, both for themselves and (potentially) their children.

### Parental attitudes regarding adolescent relationships

In Thai tradition, having premarital sex has traditionally been viewed with social disapprobation and disgrace. Premarital sex in adolescence was traditionally viewed with stigma in Thai society, but it has become increasingly normalised nowadays. This is because attitudes towards premarital sex reflect the changing nature of modern society, and such behaviours are now widely accepted in society. In previous studies, adolescent sexuality and pregnancy seemed relatively normal phenomena in rural communities in Thailand [30, 78]. Sriyasak, Almqvist [78] reinforced that the changing of the dynamic social environment becomes the accepted in the traditional value in the North-eastern of Thailand. Notwithstanding, modernity is the factor that has stimatised adolescent pregnancy made it shameful in Thailand. As can be seen in many previous studies in Thailand, pregnancy in adolescence is viewed as problematic and a serious social problem and pregnant adolescents are sensitive to being personally shamed by the words and gestures of people around them [79, 80].

As this study found that parents play a major role in SRH of adolescents in Thailand. Leekuan [30] found that adolescents initially concealed their sexual relationship and pregnancy from anybody, especially their parents because of being afraid of rejection. Dalton [81] encouraged adolescents to commonly hide sexual-related behaviour from their parents in the American context. This is in a line with Saim, Dufåker [82] who revealed that Malaysian adolescents also tried to hide their sexual relationship and their pregnancies.

Parents have always been the most important influence on adolescents’ decisions about sex in many contexts, but parents commonly underestimate the impact they have on their children’s decisions [74]. This study also found that parents know about and have confidence in their daughters’ decisions and activities. Surprisingly, this differs from a study by Anyanwu, Akinsola [83] in the South African context, which found that African adolescents were uncomfortable sharing their personal problems with their parents because they worried about their parents’ forbidding them dating. Generally, parenting tasks and responsibilities are an inherent part of raising children, and effectively promote their development. A study by Morales, Vallejo-Medina [69] in Colombia revealed adolescents believe that important people in their lives expect them to protect themselves during sexual intercourse, and most of them are willing to comply with these expectations. The relationships between parents and adolescents can also shape adolescent development [84].

Adolescents in this study recounted that their parents were concerned about sex-related issues, as well as birth control. Many previous studies presented similar findings, including parental perceptions of sexual relationships in adolescence [85]. A study by Breuner, Mattson [86] identified that adolescents’ perceptions of parental expectations about sex and contraception had an important impact on their sexual activities, including the use of contraceptive methods.

Contrary to expectations, parents are more likely to talk with their adolescents about body changes and dating, rather than have discussion about sex-related issues, birth control, and STIs [74]. International research shows that communication between adolescents and parents on issues such a sexual relationships, early pregnancy, HIV, and contraception is often very limited [69, 87, 88]. Because of a lack of knowledge and skills, as well as cultural norms and taboos concerning the discussion of sexual issues between parents and children, sexuality and sex education are commonly not discussed at home [88].

Previous research indicated that parental disapproval about adolescent sexual activity is an effective way to decrease the probability of adolescents engaging in sexually risky behaviours [89]. Nonetheless, the findings in this study illustrated that the adolescents were more mature and could take responsibility, especially as they were studying at university with parental approval for their relationships with their partners. This suggests the great importance of adolescent responsibility and choices in such contexts, as Leekuan [30] studied cases of adolescent pregnancy and reported that although parents in many families consented to their adolescents cohabiting prior to marriage, and having sexual relationships, the adolescents did not give importance to protecting themselves from unintended pregnancy.

Although some adolescents’ parents approved early premarital sexual relationships, many were concerned about these relationships. Lack of disclosure about sexual relationships in this study was due to fear of the reactions from parents and families. A study in Kenya by Maina, Ushie [90] revealed similar findings where romantic relationships were covert as they were viewed as immoral as well as disruptive towards achieving education goals. This is a line with a study by Killoren, Campione-Barr [91] which found that American adolescents would choose to disclose general thoughts and concerns related to sexual relationship to their mothers but would not discuss thoughts and concerns related to dating.

Our study has show that some adolescents are satisfied their relationship with their parents and had parental approval of their having sexual relationships and were able to have open discussion and communication around sexual health and sexual attitudes. Parental attitude is key in establishing healthy, reliable, respectful and responsible to the individual provides the adolescents a solid feeling of self-identity [92]. Indeed, the attitudes of the parents toward adolescent relationships are more important in the emotional and cognitive development of adolescence. Having an early premarital sexual relationship was not only approved by some parents’ adolescents, but it was also concerned about negative consequences of premarital sex [30, 33]. Otherwise, parental positive attitudes toward adolescents’ sexual relationships are particularly helpful to those adolescents having less parent-adolescent conflict [93]. Adolescents’ parents should gradually provide information about sex education in order for decreasing adolescents’ risk-taking of SRH [30, 33].

### Limitations and strengths

This study has some limitations that must be acknowledged. Firstly, this research has interpreted and given meanings to the experiences of sexual reproductive Health among 30 late adolescents recruited from a university in Northern Thailand. Individuals from other universities may have had different experiences and meanings, therefore the findings must be interpreted carefully. Secondly, late adolescents who participated in the study might have had different viewpoints from those who refused participation. Finally, socio-cultural differences among participants might have affected study findings.

The strength of the present study is its updating of knowledge on SRH experience among late adolescents in Thailand. Reported data provide a better insight into the meaning of SRH experiences pertaining to the risks of unplanned pregnancies and STIs in this population.

## Conclusion

To the best of our knowledge, this is the first study to explore the experiences of SRH in late adolescents in Thailand. Data indicate that SRH education and open communication about sex relationships in adolescence promotes a sense of intimacy and emotional closeness, which leads to greater wellbeing during the transition to adulthood.

Adolescence is a unique and critical phase in the lifespan, during which individuals attain sexual maturity. The current study uncovered how parental perceptions about adolescents’ sexual relationships are instrumental in preventing both unintended pregnancy and STIs. As a result of this, most participants made decisions to protect themselves with safe sex practices. Additionally, adolescent attitudes about practical, social, and moral implications of using contraception are also linked to their making the decision to use contraceptive methods. While the dimension of socio-economic status was not directly addressed in the study, the fact that participants were university undergraduates implies that they are from families with relatively higher socio-economic status, which should be considered in interpreting the findings.

Late adolescents in this study reported having had a special romantic relationship with their partners, which can support long-term relationships and promote taking responsibility. Their relationships with partners can also improve equality in emotional resources, sharing power in interaction and sharing decision-making responsibility.

Based on the understandings of SRH experience in adolescents, this study addresses the gap between SRH experiences and adolescent development. Without this understanding, health education cannot move forward in clinical or educational interventions. Understanding SRH experience among late adolescents is also useful for interventions and helping healthcare professionals to understand and provide more effective interventions, helping guide adolescents during a critical life stage.

The research findings provide insights into the SRH experiences of late adolescent university students in the context of Northern Thailand. The adolescents’ accounts underlying SRH experiences about sexual relationships indicated motivations for having SRH education. The findings uncovered how these adolescents comprehend their SRH education and made decisions to use different contraceptive methods in order to avoid the risk of STIs and unintended pregnancy, evidencing good levels of competence and confidence. The key priority is now to sustain this and also be mindful for future generations as well as other populations of late adolescents’ need- based experiences.

## Data Availability

Data available of reasonable request.
